# Silent Migration of a Left Common Iliac Venous Stent to the Right Atrium: A Case Report and Review of Literature

**DOI:** 10.7759/cureus.40310

**Published:** 2023-06-12

**Authors:** Sindhu C Pokhriyal, Myo Myint Tun, Ambika Devi Kaphle Bastola, Shwe Yee Htet, Sagar Nagpal

**Affiliations:** 1 Internal Medicine, One Brooklyn Health, New York, USA; 2 Internal Medicine, Nobel Medical College Teaching Hospital, Biratnagar, NPL; 3 Internal Medicine, Interfaith Medical Center, New York, USA; 4 Internal Medicine, University at Buffalo, Buffalo, USA

**Keywords:** complications, extraction, stent, venous, migrated

## Abstract

In the past decade, percutaneous endovenous stenting has emerged as the primary procedure for treating symptomatic venous outflow obstruction. Stent migration is a rare but serious and well-recognized complication of venous stenting. Cardiopulmonary complications following stent migration can manifest in a number of ways, including damage to the valves, arrhythmias, endocarditis, tamponade, and acute heart failure. Both extracardiac and intracardiac dislodgement of stents may be treated with catheter-directed extraction, stent redeployment, or surgical extraction. The decision on the type of procedure depends on multiple factors including the location of the stent, the size and accessibility of the stent, the symptoms, the extent of damage to the vital structures, and the overall health of the patient. We present the case of a 68-year-old male who presented with tachycardia. On further evaluation and workup, he was found to have an iliac venous stent that had migrated to the right atrium.

## Introduction

Venous stenting is generally considered a safe procedure, with its most common indication being venous occlusive disease. However, one rare but serious complication of venous stenting is stent migration, with reported incidence rates ranging from 0.9% to 4.3% [1]. The highest incidence of migration occurs in the iliocaval segments, which are the most common sites for stent placement, followed by central and renal veins [2]. Intracardiac migration of venous stents has been more commonly reported as a complication of central and renal venous stenting. However, intracardiac migration of stents can result in multiple complications, including valvulopathy, heart failure, and arrhythmia [3]. In this case report, we present a patient with a migrated iliac venous stent to the heart, which was incidentally found while working up the patient for sepsis.

## Case presentation

A 68-year-old male with a past medical history of hyperlipidemia, deep vein thrombosis (DVT) of the left popliteal vein (on rivaroxaban), bilateral common iliac vein stenosis s/p bilateral stent placement, chronic peripheral venous insufficiency, depression, dementia, catatonia with mutism, and bed bound from the nursing home was brought to the ED due to tachycardia for one day. As the patient was non-verbal at his baseline, pertinent history inquiries were futile. Upon evaluation, the patient’s blood pressure was 114/75 mmHg and heart rate was 140 beats per minute. On physical examination, the cardiovascular findings were negative for any murmurs or added sounds. However, the abdomen was noted to be soft with mild distension and non-tender and bowel sounds were normal.

**Figure 1:**
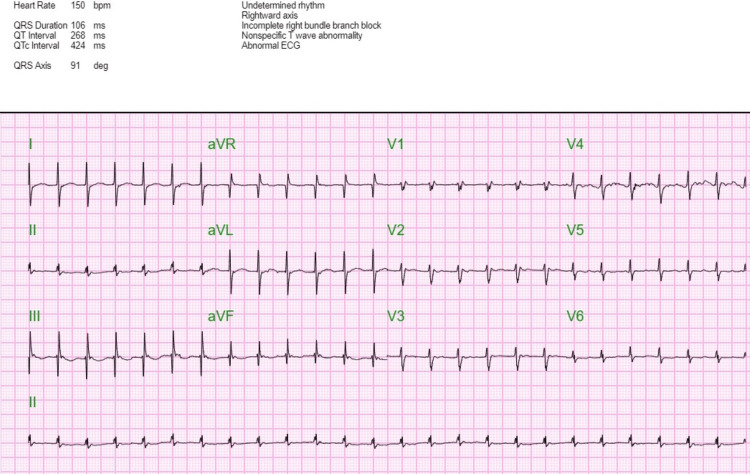
ECG showing supraventricular tachycardia with RBBB

**Figure 2:**
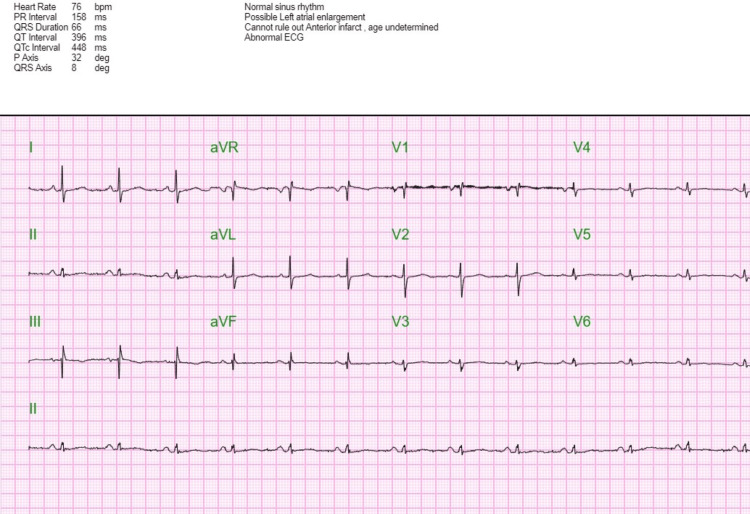
Post-adenosine ECG

ECG revealed a ventricular rate of 151 beats per minute with narrow QRS complexes consistent with supraventricular tachycardia (SVT). The episode of SVT was successfully terminated with 6 mg followed by 12 mg adenosine intravenous pushes (Figures 1, 2).

**Figure 3:**
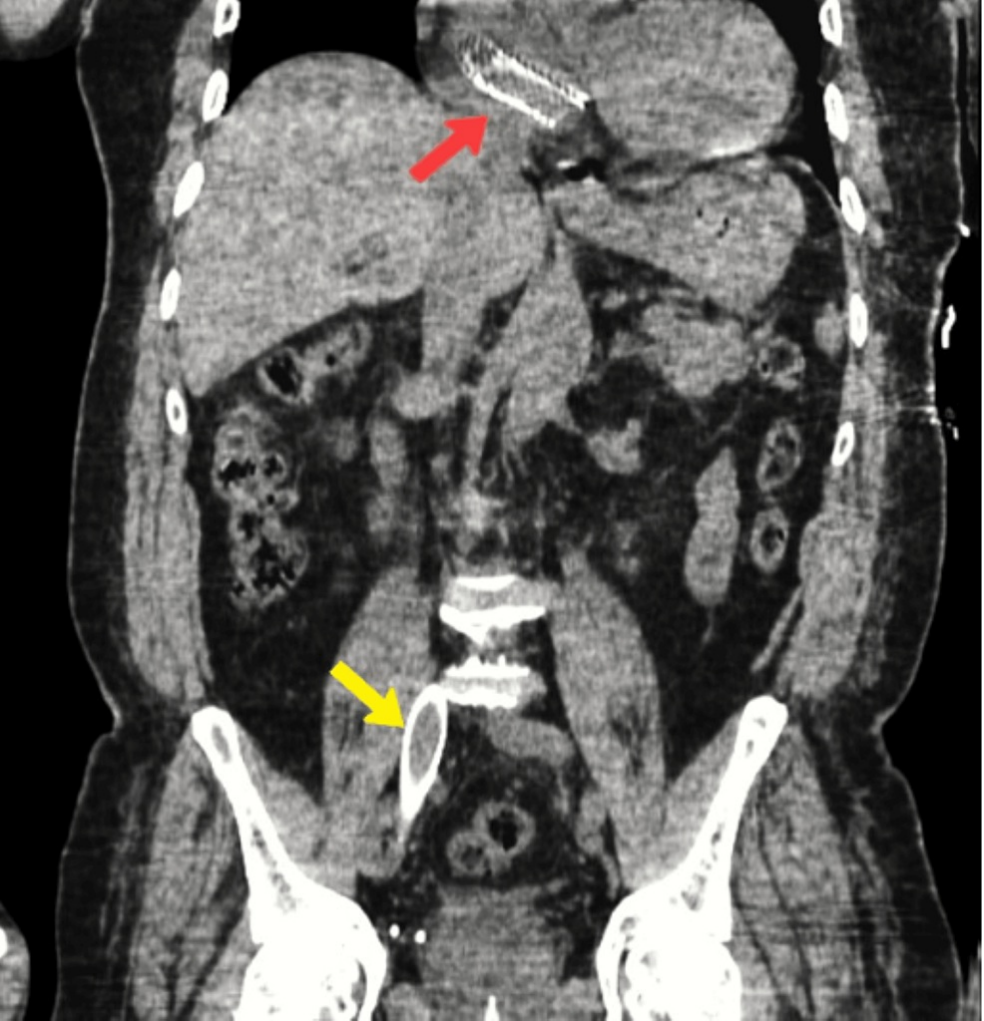
CT of the abdomen (coronal plane). Red arrow pointing at the migrated stent in the right atrium. Yellow arrow pointing at the right common iliac vein stent in place

**Figure 4:**
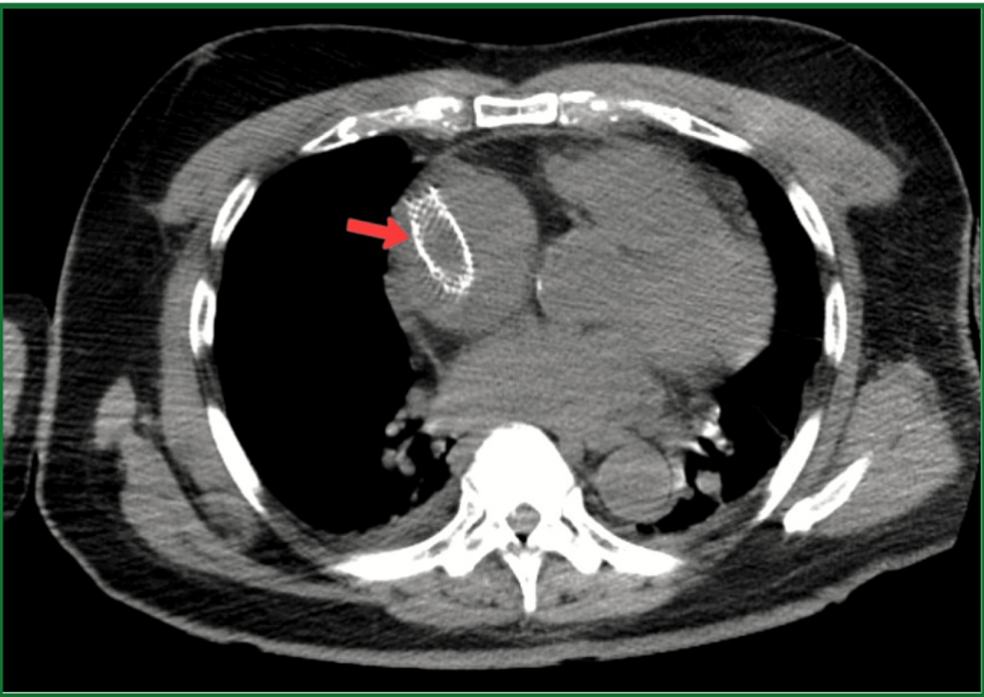
CT of the abdomen (transverse plane). Red arrow pointing at a stent in the right atrium

Labs were remarkable for WBC of 17000 cu mm, and urinalysis revealed leukocytes, nitrate, and moderate WBCs. All other labs done were normal. The patient was admitted with suspected urosepsis, and ceftriaxone 1 gm iv every 24 hours was started. A urine culture on day 2 of admission grew *Escherichia coli* (*E. Coli*). A CT scan of the abdomen and pelvis was performed to assess intra-abdominal pathology for distention and obstructive pathology for complicated UTI. There was no obstructive pathology in the urinary tract but identified two stents: one in the right atrium and one in the right common iliac vein (Figures 3, 4).

**Figure 5:**
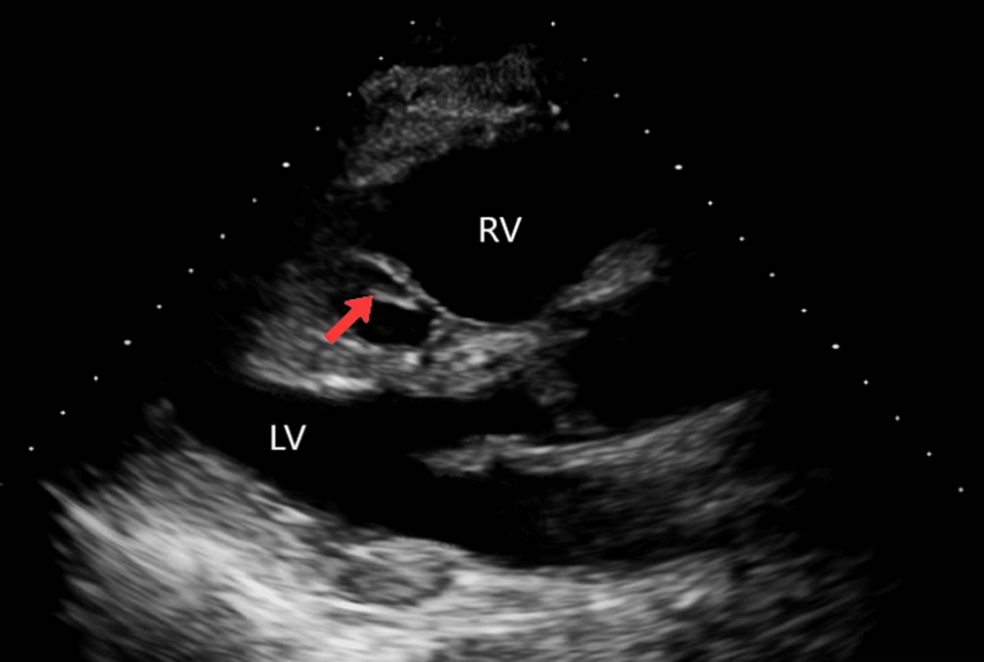
Parasternal short axis view in transthoracic echocardiogram with red arrow pointing at a tubular structure representing the venous stent

Pertinent history obtained from the caretakers and the next of kin (NOK) revealed a history of stenosis in the right and left common iliac veins, followed by stent placement in December 2022. The venous stents were noted as 16 mm x 100 mm and 18 x 80 mm stents to the left and right common iliac veins, respectively. At this point, it was confirmed that one of the stents had dislodged and migrated into the right atrium. The dislodged stent was again noted in the transthoracic echocardiogram which described it as a tubular mass in the right atrium (Figure 5).

The patient's hospital course was uneventful with regard to arrhythmias during telemetry monitoring, and he was successfully treated for *E. coli* UTI. Rivaroxaban was initiated as recommended by the hematology team to mitigate the risk of thromboembolism associated with the dislodged stent located in an unusual position. After a multidisciplinary meeting involving specialists from cardiology, vascular surgery, and hematology, the patient was transferred to a higher-level facility for stent retrieval. At the transferred facility, a transesophageal echocardiogram was performed for a detailed localization of the migrated stent spotting the distal end at the IVC-RA junction, the proximal end at the right atrial appendage. Finally, an attempt was made to percutaneously extract the stent using a snare, but it was unsuccessful due to the stent being embedded in the atrial wall. Given the patient's poor surgical candidacy, multiple discussions were held with the next of kin (NOK) and the specialties team, and a conservative approach was chosen instead of pursuing open-heart surgery. As a result, almost nine days after the initial presentation, the patient was discharged back to the nursing home with an oral anticoagulant to prevent clot formation within the stent.

## Discussion

Venous stenting is increasingly being used as the interventional treatment modality for central and peripheral venous occlusion/stenosis or compression. It has also been used for intentionally creating shunts, e.g., intrahepatic portosystemic stent shunts, or in arteriovenous fistula for hemodialysis access [4,5].

While it is generally considered a safe procedure, there are several adverse outcomes associated with it during or after the placement of the stent [1]. Misplacement can lead to trauma to the nerves or veins, while migration after the placement can have worse outcomes. While we focused on the femoral and iliac veins pertinent to our case, stent migration can occur in any vein, including iliac veins, femoral veins, innominate veins, brachiocephalic veins, and subclavian veins [4,5].

Stent migration is an uncommon complication of the procedure. A few case articles included prior history of massage or trauma before the stent migration and several factors that influence the migration of the stent. These factors include the size of the stent, the location of the stent, and the diameter of the vessel [6]. Upper extremity venous stent migration is less common as compared to iliac and renal stents. Shorter stents of less than 60 mm and smaller diameters of less than 14 mm are one of the common variables seen in our review of the literature associated with an increased risk of stent migration [2,7,8]. Central veins with larger diameters have a higher chance of migration. It is also believed that dynamic variation of the venous diameter depending on the venous return is one of the contributing factors for stent migration [9,10].

Based on the literature review, we found that stent migration can occur immediately following the procedure or can occur after many years [9,10,11]. Stent migration can be incidentally discovered without any signs and symptoms but can also present with varying symptoms depending on the site of the dislodgement. We found that the common presentations included chest pain, dyspnea, or exertional shortness of breath [6,10]. Subtle clinical clues could be found in the ECG showing findings of bradycardia, atrial flutter, non-sustained ventricular tachycardia, atrioventricular block, and or myocardial infarction [11]. Imaging aids in confirming and localizing the migrated stents. CT of the chest and echocardiogram are very valuable for the assessment as well as planning for further management.

In the current review, we learned that the most frequently migrated sites are the right side of the heart including the right atrium, right ventricle, and infrequently to the pulmonary artery with involvement of the nearby structures including the atrioventricular septum and tricuspid valves which can present with murmurs as well [10,12,13]. With the exception of one instance of mortality prior to a thorough assessment, we found that the clinical presentation and severity of each of these cases are variable. The majority of cases of stent migration could be managed with specialized cardiothoracic surgery, vascular surgery, and/or the interventional radiology team [5,6,11].

As per the literature, the management of the migrated stents depends on the severity of the presentation. In a few cases, especially with asymptomatic ones, patients were treated conservatively with follow-up chest X-rays, clinical clues, and ECG [2]. While a less invasive form of percutaneous endovascular retrieval seems very helpful, there were several incidents where this approach was not only unsuccessful but also led to fragmentation of the stent and injury to the valves [6]. Even in our case, we attempted the endoscopic approach without success as the stent remained embedded in the right atrial wall. Open surgical intervention was successful in the removal of the stents and provided an opportunity to fix the damaged structures (valves, coronaries, septum). While not applicable to our case, few cases have repositioned the stents in the correct place using balloon angioplasty without removing it [9].

The attached Table 1 summarizes the common indications, demographics, and other variables we used in the literature review focusing on the iliofemoral venous stent and other factors related to stent migration.

**Table 1:** Summary of the common indications, demographics, and other variables used in the literature review focusing on the iliofemoral venous stent and other factors related to stent migration SVT: supraventricular tachycardia, CXR: chest X-ray

Index	Date/year	Age in years/gender	Indication/stent	Duration after placement/size of the stent	Presentation	Migrated to	Outcome
Current case	2022	68/Male	Bilateral common iliac vein stenosis	3 months. Left 16 x 10 0mm. Right 18 x 80 mm	Tachycardia/SVT	Right atrium	Survived/failed endoscopic intervention
1	2022 [3]	49/Male	Chronic venous stasis with left iliac vein occlusion	10 months. 16 × 60 mm	Chest pain	Right atrium/tricuspid valve	Survived/surgical removal with valve repair after failed endovascular retrieval
2	2018 [4]	20/Female	May-Thurner syndrome	Few days	Leg pain/paresthesia/paresis	Spinal canal L4 foramen	Survived/surgical removal
3	2009 [5]	78/Male	Severe stenosis in left innominate vein	2 days	General fatigue and bradycardia	Right ventricle	Survived/surgical removal after failed percutaneous endovascular extraction
4	2008 [6]	53/Male	Postphlebitic syndrome due to infrainguinal deep vein thrombosis/femoral vein	3 years	Incidental finding on the CXR for another condition	Right atrium	Survived/surgical removal after failed percutaneous endovascular extraction
5	2020 [9]	59/Male	May-Thurner syndrome or iliac vein compression syndrome ( 90% occlusion of left common and external iliac veins)	3 days	Dyspnea/diaphoresis/inferior wall myocardial infarction	Right atrium with perforation to Rt coronaries/perforation of myocardium	Survived/surgical removal/valve repair
6	2017 [10]	55/Female	Uncertain-stent below inferior vena cava	1 day	Chest pain	Right ventricle	Expired even before any intervention
7	2006 [11]	53/Female	Stent for the Iliac vein stenosis	1 year	Non-sustained ventricular tachycardia and moderate tricuspid regurgitation	Apex of the right ventricle	Survived/surgical removal after failed percutaneous endovascular extraction
8	2020 [12]	56/Female	Deep venous thrombosis of the right external iliac vein	3 years	Dry cough/fever	Right pulmonary artery	Survived/surgical removal/valve repair
9	2022 [13]	74/Female	May-Thurner Syndrome/B/L common Iliac vein	5 years	Dyspnea/arrhythmia (atrial fibrillation, supraventricular tachycardia)	Right ventricular outflow/right interlobar pulmonary artery	Survived/surgical removal of right ventricular stent
10	2022 [14]	70/Male	End-stage renal disease/arteriovenous fistula	1 year. 8 x 20 mm	Incidental	Right ventricular	Survived/conservative management
11	2018 [15]	46/Female	May-Thurner syndrome	Days-4 months. 14 × 60 mm	Chest discomfort	Right ventricular outflow	Survived/surgical removal with valve repair after failed endovascular retrieval
12	2018 [16]	61/Female	May-Thurner syndrome with left iliac vein stent	6 months	Lower extremity edema, shortness of breath, orthopnea	Tricuspid valve from the right atrium	Survived/surgical removal with valve repair after failed endovascular retrieval where snared segment fractured
13	2017 [17]	61/Male	Right iliac vein stenosis	3 days/10 x 60mm, 10/40 mm	Fever, pneumonia	Right ventricle	Open surgical approach
14	2011 [18]	40/Male	Subclavian stenosis in HD patient	Right after procedure	No immediate symptoms, dyspnea on exertion after 3 months	Right ventricular apex	Survived/surgical removal with valve repair after failed endovascular retrieval
15	2005 [19]	55/Male	Inferior vena caval filter	2 years	Tachycardia	Right atrium	Survived/endoscopically
16	2002 [20]	32/Male	Right iliac stenosis/obstruction	2 months	Incidental finding on the routine chest X-ray later developed chest pain	Right ventricular and pulmonary artery	Survived/percutaneous removal

## Conclusions

Venous stent migration is an infrequent but potentially fatal complication of stent placement. It can present asymptomatically or with subtle nonspecific clinical signs and symptoms such as shortness of breath, chest pain, bradycardia, and arrhythmias. With growing trends of stent deployment, clinicians should keep a high level of suspicion for the diagnosis of stent migration and plan for urgent intervention including open heart surgery and the endoscopic approach depending on the acuity of the situation.
